# Heat Shock Improves Random Spore Analysis in Diverse Strains of *Saccharomyces cerevisiae*

**DOI:** 10.3389/fgene.2020.597482

**Published:** 2020-12-11

**Authors:** Molly K. Burke, Kaitlin M. McHugh, Ian C. Kutch

**Affiliations:** Department of Integrative Biology, Oregon State University, Corvallis, OR, United States

**Keywords:** random spore analysis, heat shock, sporulation, genomics, multiparent mapping population

## Abstract

Random spore analysis (RSA) is a classic method in yeast genetics that allows high-throughput purification of recombinant haploid spores following specific crosses. RSA typically involves a number of steps to induce sporulation, purge vegetative cells that fail to sporulate, and disrupt the ascus walls of sporulated cells to release haploid spores. These steps generally require expensive chemicals and/or enzymes that kill diploid cells but have few effects on spores. In the fission yeast *Schizosaccharomcyes pombe*, heat shock has been reported as an effective addition to RSA protocols, but to our knowledge heat shock has not been used for this purpose in the budding yeast *Saccharomyces cerevisiae*. Here, we evaluate the effects of heat shock on vegetative and sporulated cultures of four diverse yeast strains: a European wine strain (DBVPG6765), a Japanese sake strain (Y12), a West African palm wine strain (DBVPG6044) and a North American strain isolated from the soil beneath an oak tree (YPS128). We characterize this phenotype under multiple combinations of temperature and incubation time, and find specific conditions that lead to the exclusion of vegetative cells and an enrichment in spores, which differ by strain. We also collected genome sequence data from a recombinant population that experienced multiple rounds of RSA, including one round with a heat shock treatment. These data suggest that when incorporated into an RSA protocol, heat shock leads to increased genetic diversity among the cells that survive and mate. Ultimately, our work provides evidence that short heat treatments can improve existing RSA protocols, though in a strain-specific manner. This result informs applications of high-throughput RSA protocols, such as QTL mapping and experimental evolution research.

## Introduction

Many traditional methods in genetics, including linkage mapping and quantitative trait locus (QTL) mapping, require controlled crossing of laboratory organisms followed by screening recombinant progeny. Ascomycete yeasts are especially valuable model systems in this context, as diploid cells produce four viable haploid spores (a “tetrad”) via meiosis that can be physically dissected and scored to analyze patterns of segregation. This so-called tetrad analysis (Fincham et al., 1979) is relatively low-throughput and requires skilled technicians and specialized equipment (Sherman and Hicks, 1991). Random spore analysis (RSA) is an alternative, higher-throughput approach for screening large numbers of recombinant progeny (Bevan and Woods, 1963). The general strategy behind RSA involves: (i) generating a large culture of cells that have mated then sporulated *en masse*; (ii) selectively killing any vegetative diploids that fail to sporulate; and (iii) releasing individual spores from asci. This technique produces millions of spores with unique genotypes from a single genetic cross which can be characterized at phenotypic and genetic levels.

While RSA has been a classic technique in yeast genetics for decades, recent years have brought renewed interest in improving this method for modern genomics applications. QTL mapping in the yeast *Saccharomyces cerevisiae* historically involves pairwise crossing between two genetically distinct laboratory strains, and while this effort has been fruitful (e.g., Steinmetz et al., 2002; Brem et al., 2005; Bloom et al., 2013), it neglects a great deal of potentially functional variation segregating in natural populations. As more diverse natural isolates are cataloged and made amenable to genetic interventions (e.g., Cubillos et al., 2009), multiple investigators have established so-called “multiparent mapping populations” by crossing strains from various geographical origins. Cubillos et al. (2013) created a 4-parent population, and Linder et al. (2020) created an 18-parent population, as community resources for mapping complex traits in yeast. While including more parental strains in a mapping population increases its capacity for trait mapping, it also requires random spore analysis, as tetrad dissection becomes prohibitively laborious. In related efforts to map complex traits in yeast, investigators have also become increasingly interested in using random spore analysis in experimental evolution studies. Experimental evolution generally involves subjecting a laboratory population to specific stressors over generations and characterizing the phenotypic and genomic changes that result (reviewed by Long et al., 2015). In yeast, experimental evolution can either involve initially clonal populations that accumulate *de novo* mutations (e.g., Lang et al., 2013), or initially recombinant populations in which natural selection acts upon segregating variation (Burke et al., 2014; Kosheleva and Desai, 2018). The latter approach is becoming more popular, especially as multiparent mapping populations, which by design harbor extensive segregating variation, are being generated and distributed within the community. In order to optimally map traits using experimental evolution in recombinant populations, random spore analysis is again needed. As an example, Burke et al. (2014) subjected large multiparent populations to 18 weeks of a sustained selection regime, and induced random spore analysis once per week to shuffle genotypes and maintain genomic variation in the populations. This strategy successfully identified small (∼10 kb) regions of the genome that could be confidently associated with the traits under selection.

As diverse *S. cerevisiae* strains become more deeply phenotypically and genomically characterized (e.g., Peter et al., 2018), we expect studies of outcrossing yeast (i.e., multiparent mapping populations and outcrossing experimental evolution) to increase, which will in turn increase demand for efficient and high-throughput protocols for random spore analysis. Notably, random spore analysis generally requires costly or caustic reagents. Procedures for enriching yeast cultures for sporulated cells typically exploit the differential sensitivity of spores and vegetative cells to stressful conditions. There are numerous techniques for accomplishing this, which tend to involve caustic reagents such as diethyl ether that kill vegetative cells (Dawes and Hardie, 1974; Parenti-Castelli et al., 1974), and expensive lytic enzymes that weaken ascus walls and physically break up spores of a tetrad (Sherman, 2002). Due to the general effectiveness and robustness of these techniques, laboratories tend to develop specific in-house protocols for RSA based on their specific needs and resources, though some studies have examined ways to optimize efficiency and throughput (e.g., Bahalul et al., 2010).

In the fission yeast *Schizosaccharomyces pombe*, spores are more resistant to heat shock than vegetative cells, and the use of heat has been demonstrated as an inexpensive and non-toxic method for enriching cultures for cells which have successfully sporulated (Khare et al., 2011). Khare et al. (2011) show that exposing a mixed culture of asci and vegetative cells to 55°C for 10–30 min dramatically decreases viability in vegetative cells with little impact on spores. This study showed that UV stress and freeze/thaw stress can also selectively kill vegetative cells, but at significant cost to spore viability; thus, the authors conclude that heat shock can be incorporated into RSA protocols to increase efficacy and decrease cost.

Here, we examine the potential for heat shock to add value to RSA protocols in the budding yeast *S. cerevisiae*. Following the general design of Khare et al. (2011), we subject cultures containing either purely vegetative cells, or mixes of sporulated and vegetative cells, to a number of conditions of heat shock intensity and duration. We carry out these assessments in four geographically and genetically distinct strains, each from a different continent—three fermentation strains and one wild isolate. We also create a 4-parent recombinant population from these strains, and impose repeated cycles of sporulation, random spore isolation and mating. We collect whole-genome sequence data from this unreplicated population initially, after six cycles, and after 12 cycles of this treatment, and the latter timepoint includes a replicate implementing 20 min of heat shock at 55°C. Our results generally suggest that heat shock can be a useful addition to RSA protocols in *S. cerevisiae*, but that the particular temperature and duration of heat shock stress that results in optimal spore enrichment varies depending on strain.

## Materials and Methods

### Haploid and Diploid Isogenic Strains

Strains used this study belong to the Saccharomyces Genome Resequencing Project (SGRP; Liti et al., 2009), a collection of isolates from around the globe that have been tagged with unique genetic barcodes, and made heterothallic so they can be easily crossed (Cubillos et al., 2009). We focus on four of these strains, listed in Table 1, which have been previously phenotypically characterized (Warringer et al., 2011), and extensively genome sequenced (e.g., Bergström et al., 2014; Linder et al., 2020). Linder et al. (2020) further modified these haploid strains so that *MATa* and *MAT*α haploids have specific drug resistance markers linked to the mating-type locus, so that *MATa* strains can be recovered in media supplemented with 100 μg/mL nourseothricin sulfate (“NTC”), *MAT*α strains can be recovered in media supplemented with 300 μg/mL hygromycin B (“hyg”), and mated *a*/α diploids can be recovered in media containing both drugs. For the initial phenotypic characterization of each strain following heat shock, *a*/α diploids were created by streaking cultures of each haploid strain in a cross shape on YPD plates (so that mating types came into contact where the streaks cross), allowing growth at 30°C for 48 h, and replica plating onto YPD/NTC plates and YPD/hyg plates. After another 48 h of growth at 30°C, the resulting diploid colonies were sampled and streaked onto fresh YPD/NTC/hyg plates twice to ensure that the only a single colony was selected. These colonies were grown overnight in YPD and archived for future use.

**TABLE 1:** Strains used in this study, their geographical origins, and sporulation efficiencies after ∼72 h.

Strain name	Origin	Sporulation efficiency (mean of *n* = 3 ± SD)
YPS128	United States; soil beneath *Quercus alba*	0.669 ± 0.048
4X	Recombinant	0.632 ± 0.02
Y12	Japan; sake	0.491 ± 0.032
DBVPG6044	West Africa; palm wine	0.029 ± 0.025
DBVPG6765	Europe; wine	0.026 ± 0.0189

*Reported sporulation efficiencies are the means of three biological replicates (independent sporulating cultures) ± one standard deviation of the mean. P-values from post-hoc Dunn tests are provided in Supplementary Table S4.*

### Creation and Maintenance of a 4-Parent Cross

For the genomic characterization of recombinant populations that go through RSA protocols with or without a heat shock step, we first generated a 4-parent population (“4X”) by crossing the haploid strains listed in Table 1. Haploid cultures of individual strains were streaked as described above to obtain mated *a*/α diploids, this time crossing the different strains; DBVPG6765 (*MAT*a) was crossed to YPS128 (*MAT*α), and DBVPG6044 (*MAT*a) was crossed to Y12 (*MAT*α). The diploid colonies recovered on YPD/NTC/hyg agar plates were transferred to 1 mL of liquid YPD, and grown overnight in the shaking incubator (30°C/200 rpm) to increase cell numbers. Cells were then collected, spun down, washed with sterile H_2_O, and resuspended in 1 mL minimal sporulation media (10 g potassium acetate/L H_2_O); cells then were allowed to sporulate for 3 days in the shaking incubator (30°C/200 rpm). Tetrads from these diploid cells were dissected using a SporePlay tetrad dissecting microscope (Singer) and arrayed onto YPD plates. The four haploid spores of each tetrad were replica plated onto YPD plates containing either NTC or hyg to verify the proper 2:2 segregation of drug resistance markers and thus mating types. Each of the four validated (now recombinant) haploid colonies per cross were grown overnight in 1 mL YPD to increase numbers, then pooled in equal proportions in a single 50 mL conical tube. These cells were washed and resuspended in 8 mL fresh YPD media, then cells were left at room temperature for 90 min to settle into a loose pellet and begin to mate. After this time, ∼200 μL aliquots of this mating mix were spread onto 10 YPD/NTC/hyg plates and allowed to grow for 48 h at 30°C. These mated diploid cells were then scraped off agar plates with a sterile glass slide, transferred to fresh YPD media, and archived as the “ancestral” 4X population.

The 4X population was then taken through 12 cycles of outcrossing, using a protocol implementing various techniques for random spore isolation adapted from methods used in previous work (Burke et al., 2014) as well as by Linder et al. (2020). Briefly, newly mated diploid cells were grown overnight in 10 mL YPD media. Saturated cells were spun down and washed with sterile H_2_O, then resuspended in 40 mL sporulation media in a 250 mL Erlenmeyer flask for a 72 h sporulation period in the shaking incubator (30°C/200 rpm). Cells were then collected, spun down into pellets, washed with sterile H_2_O, and resuspended in Y-PER yeast protein extraction reagent (Thermo) for 20 min; this reagent kills vegetative (unsporulated) diploids in a manner similar to diethyl ether, with little impact on spore viability. Cells were again spun down, supernatant aspirated off, and resuspended in a reagent cocktail containing 1% zymolyase (Zymo Research) to weaken ascus walls, as well as 0.5 mm silica beads (BioSpec) to mechanically agitate the asci; cells were then vortexed at maximum speed for 6 min. Cells were spun down a final time, washed in sterile H_2_O, resuspended in 10 mL YPD and allowed to settle for 90 min at room temperature, during which time cells begin to mate. This mating culture was then transferred to 10 individual YPD/NTC/hyg agar plates in 200 μL aliquots (remaining cells were discarded) and allowed to recover for 48 h at 30°C. The resulting lawns of mated diploid cells were collected by scraping with a sterile glass slide into a fresh 50 mL conical tube of YPD; this “cell bank” was sampled for archiving at −80°C, it was sampled for DNA extraction, and it was also used to initiate an overnight culture for the next outcrossing cycle. After the 11th cycle, two replicate “cycle 12” cultures were split/initiated, and during the cycle 12 outcrossing protocol, one replicate received the regular treatment, while the other (cycle “12 H”) received a heat shock incubation step of 55°C for 20 min in a dry bath. This heat shock step occurred following the initial washing of spores in sterile H_2_O, immediately prior to resuspension in Y-PER. All other steps of the protocol were included as usual.

We estimate that ∼15–20 cell doublings occur between every outcrossing cycle of the experiment. Based on counting colonies from dilutions of cultures plated at various benchmarks during the protocol, we expect that a range of 7.5–11 generations occurs during the period of diploid recovery on agar plates, and that a similar range of generations occurs during the overnight culture in YPD media. Therefore, over the 12 cycles of outcrossing there has likely been at least 15^∗^12 = 180 generations of evolution.

### Heat Shock Viability Phenotyping

All diploid strains in Table 1, as well as a 4-parent cross between these strains, were assessed for cell viability following heat shock in specific ways. When testing diploid strains, overnight cultures were always inoculated with single colonies, while when testing the 4-parent cross, hundreds of colonies were sampled (by scraping lawns with a wooden applicator) to preserve the genetic diversity in that cross. First, we tested the susceptibility of vegetative (unsporulated) cells to heat shock by subjecting samples of 24 h overnight cultures to 15 different temperature/incubation times following the design in Khare et al. (2011). The temperatures ranged from 40 to 60°C in 5° increments, and incubation times were 10, 20, and 30 min. Overnight cultures were diluted to a standard OD_600_ = 0.1 (± 0.01) and 1 mL of this diluted culture was aliquoted into each of 16 experimental 1.5 mL microcentrifuge tubes. One tube was allowed to sit at room temperature for 30 min as a control, and the remaining 15 tubes were placed in their assigned incubation temperatures in a dry bath for their assigned incubation times. Fifty microliters of a 1/100 dilution of these tubes were then plated onto each of five YPD plates (technical replicates); this dilution and volume generally resulted in a countable range of colonies (100–200). Mean colony counts from the five control plates were used as a baseline expectation for the total number of viable cells in the culture, i.e., 100% viability. Colony counts from the five technical replicates from each heat shock condition were then compared to this expected number to estimate cell viability, ranging from 0 to 1, as the proportion of cells that survived.

To test the susceptibility of sporulated cells to heat shock, 10 mL of saturated culture from a 24 h overnight culture of each strain was collected, washed in sterile H_2_O, and resuspended at 25% concentration in sporulation media (40 mL in a 250 mL Erlenmeyer flask). After 72 h of sporulation at 30°C/200 rpm, the mixed culture of vegetative and sporulated cells was collected, diluted to an OD_600_ = 0.1 (±0.01), and assessed in an identical manner as the purely vegetative cultures explained above. Means from the control plates were again used to convert colony counts into viability estimates for each strain. By comparing the viabilities of vegetative and sporulated cells within a given heat shock treatment, we aimed to identify heat shock conditions for optimally enriching spores; in other words, we were interested in identifying treatments that resulted in the lowest viability for vegetative cells and the highest viability for sporulated cells. Due to the non-parametric nature of the viability estimates, we used Friedman tests to evaluate the null hypothesis that the distributions of temperature-specific viabilities for each strain are equal. Tests were run separately for vegetative cultures and sporulated cultures. These tests allowed us to determine evidence for systematic differences in the distribution of viabilities across all heat-shock conditions for the strains (each temperature/incubation treatment was considered one of 15 independent blocks). A *post-hoc* procedure is necessary to provide evidence for differences between specific strains; we used the Conover test which makes multiple pairwise comparisons for rank-sum differences (*p*-values were adjusted by the “fdr” method to control the false discovery rate for multiple comparisons).

### Sporulation Efficiency Estimation

All diploid strains were assessed for sporulation efficiency in triplicate (Table 1). While prior studies also report strain-specific sporulation efficiencies, this phenotype is notoriously sensitive to preculture conditions; thus it is prudent to measure under the conditions imposed by our experiments. For each strain, multiple overnight cultures (10 mL in YPD) were initiated, such that the three cultures with the most consistent OD600 estimates after ∼24 h (1:100 dilutions were 0.1 ± 0.03) were retained for sporulation. Cells were spun down, washed twice with sterile water, and resuspended in 40 mL minimal sporulation media (1% potassium acetate). Cultures were then transferred into 250 mL Erlenmeyer flasks and incubated for ∼72 h (30°C/200 rpm). Cells were counted under 40× magnification on a SporePlay (Singer) microscope using a hemocytometer. A minimum of 200 cells were counted per replicate, and sporulation efficiencies were calculated as the proportion of observed tetrads over the total number of observed cells. Kruskal-Wallis tests were used to determine a significant effect of strain on sporulation efficiency. Subsequent Dunn tests allowed for *post-hoc* pairwise comparisons of strains.

### DNA Preparation and Genome Sequencing

At cycles 0, 6, 12, and 12 H, DNA was extracted following diploid recovery. At the same time, we also extracted DNA from the four haploid parental founder strains, using cultures inoculated from a single colony. Gentra Puregene Yeast/Bact kits (QIAGEN) were used to purify DNA from cells in late log-phase. Genomic DNA libraries were prepared using the Nextera DNA Sample Preparation Kit (Illumina) with some modifications to the standard protocol to increase throughput, including custom barcodes, smaller reaction sizes, and additional size-selection steps (similar to Baym et al., 2015; details available upon request). Libraries were individually dual-indexed, pooled at equal molarities, and run on SE150 lanes of the HiSeq3000 OSU’s Center for Genome Research and Biocomputing. Approximately 30–40 million reads were achieved for each of the four recombinant populations, and approximately 10–20 million reads were generated for each of the four isogenic strains. This discrepancy was by design as lower coverage should still provide confident allele calls in an isogenic/haploid sample.

Reads were processed using a custom pipeline that estimates allele frequencies in each population directly from sequence data; essentially, since DNA was extracted *en masse* from recombinant populations, SNP calling tools can be used to convert read counts to allele frequencies at SNP sites. We used GATK v4.0 (McKenna et al., 2010; Poplin et al., 2018) to align raw data to the *S. cerevisiae* S288C reference genome (R64-2-1) with bwa-mem and create a single VCF file for all variants identified across all four libraries. We also used a publicly available VCF file with SNP information for the strains (Bergström et al., 2014) to “train” GATK; this is a recommended best practice for calibrating base quality with GATK v4.0. The resulting VCF file was converted into a SNP frequency table with a custom python script (available upon request) suitable for downstream analysis with R^1^. SNPs were only called at sites known to segregate among the four haploid founder strains used in the 4X cross. As a final quality control measure, any sites with < 10X coverage at any SNP position were excluded; coverage in this context is the denominator in our estimate of allele frequency in each population. Using these estimated SNP frequencies, we characterized general patterns of genetic diversity in the recombinant populations by generating site-frequency spectra.

### Fisher’s Exact Tests

To determine what regions might be responding to selection during the 12 cycles of outcrossing, or as a result of the heat-shock step, we conducted Fisher’s Exact Tests (FET) at every SNP. At each of the 109,707 total SNPs, we used fisher.test() in R, making comparisons between two datasets: cycle 0 vs. cycle 12, and cycle 12 vs. cycle 12 H. The resulting *p*-values were -log(10) transformed and smoothed by averaging across overlapping groups of 50 SNPs (there is about 1 SNP every 120 bp, so 50 SNPs ∼6 kb). This choice of 50 SNPs was somewhat arbitrary, but seemed like an appropriate level of smoothing after looking at multiple window sizes and finding that this left all major peaks intact, while minimizing noise. While we acknowledge that there are many superior metrics to FET tests in evolve-and-resequence work (e.g., Vlachos et al., 2019), most of these approaches were not available to us given our lack of replication. But there is precedent in the literature for this type of analysis (e.g., Burke et al., 2010), and it provides a course picture of regions of the genome that are differentiated between two populations. To establish rough significance thresholds for each comparison, we made Q–Q plots comparing observed *p*-values against expected null values and identified the point at which values began to diverge from the line of best fit.

### Haplotype Estimation

Because we sequenced all parental strains of the 4-way cross in addition to that cross, we were able to characterize genome-wide haplotype frequencies from our pooled sequencing data. We were particularly interested in whether the heat shock step incorporated during cycle 12 of the outcrossing scheme led to over- or underrepresentation of particular haplotypes. To address this question, we estimated genome-wide haplotype frequencies in the recombinant population following 12 outcrossing cycles, with and without the heat-shock step. Haplotype frequencies were estimated using the sliding window haplotype caller described in Linder et al. (2020) and software the authors have made available as a community resource: https://github.com/tdlong/yeast_SNP-HAP. With the haplotyper.limSolve.code.R script, we estimated haplotype frequencies across 30 kb windows with a 1 kb step size.

## Results

### Heat Shock Phenotypes

**FIGURE 1:**
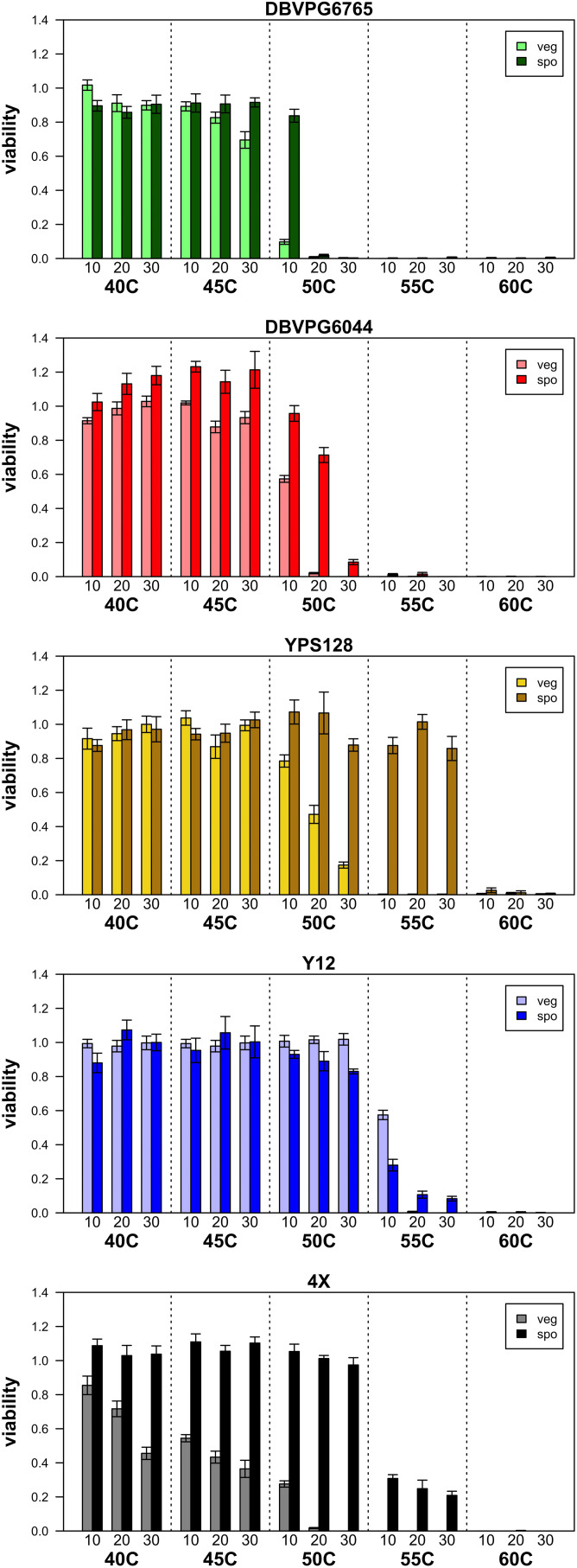
Summary of viabilities of purely vegetative cultures (light colored bars) and sporulated cultures (dark colored bars) for the four isogenic strains we assessed, in addition to the population made by crossing all four. Error bars represent SEM across five technical replicates.

Figure 1 presents a summary of the viability estimated in purely vegetative and sporulated cultures under various heat shock conditions, and Supplementary Table S1 lists the mean viabilities plotted in this figure. As the temperature and duration of heat shock increase, viability decreases both culture types, and in all strains. Generally, we find that sporulated cultures have higher overall viabilities than purely vegetative cultures, a qualitative pattern consistent with the idea that spores are more stress-resistant than vegetative cells. While Figure 1 presents data from only one biological replicate per strain, we assayed additional biological replicates of vegetative and sporulated cultures at 50°C and found remarkable consistencies in viability across replicates and assays (Supplementary Table S2).

Considering the vegetative cultures, a Friedman test returned *p* = 6.254 × 10^–07^ for the effect of strain on heat-shock viability. Kendell’s W (coefficient of concordance) can be used as an effect size statistic for a Friedman’s test, and was 0.573 (indicating a large effect size). *Post-hoc* pairwise Conover tests indicated that all strains had statistically different distributions except for YPS128 and Y12 (which had the highest overall viabilities). Supplementary Table S3A provides the *p*-values from these Conover pairwise tests. For the sporulated cultures, a Friedman test returned a *p* = 0.004569 for the effect of strain on heat-shock viability, and Kendell’s W = 0.251, indicating a medium effect size. *Post-hoc* pairwise Conover tests indicated that several strains had overlapping distributions; YPS128 showed the highest overall viabilities and DBVPG6765 showed the lowest overall viabilities, and the remaining three strains had intermediate viability values whose distributions were not significantly different (the 4X population could also not be statistically differentiated from strain YPS128). Supplementary Table S3B provides *p*-values from these Conover pairwise tests.

### Sporulation Efficiency

Estimates of sporulation efficiency are provided, in decreasing rank order, in Table 1. A Kruskal-Wallis test showed a significant effect of strain on sporulation efficiency (*p* = 0.002326). *Post-hoc* Dunn tests for multiple pairwise comparisons indicate two statistically significant groups: “high” (YPS128, 4X) and “low” (DBVPG6044, DBVPG6765) sporulating strains (Supplementary Table S4). Strain Y12 was not statistically distinct from either of these groups, which is surprising given the trait values, but suggests that our test is underpowered (due to low sample size) to detect true differences between treatments. Qualitatively, it seems clear that the Y12 strain sporulates much more efficiently than either of the low strains.

### Pooled Population Genome Data

**FIGURE 2:**
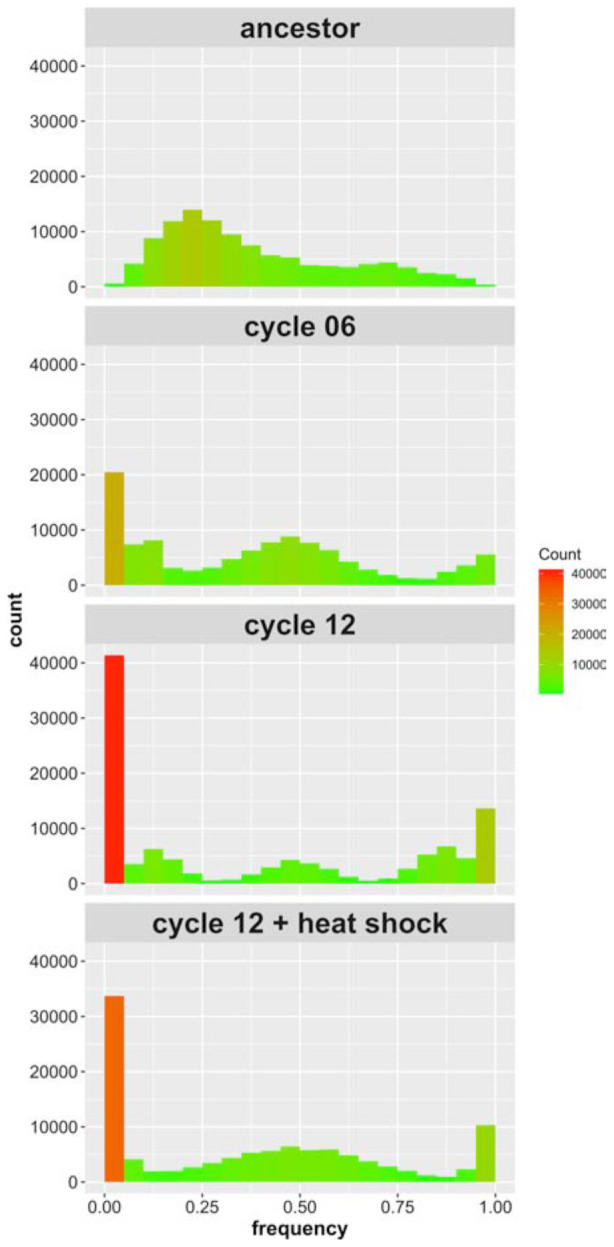
Site frequency spectrum of SNPs identified in the 4-parent population initially, after six outcrossing events, after 12 outcrossing events, and after 12 outcrossing events + a heat shock of 55°C for 20 min. Color in included in the histograms to emphasize the bins with high counts (red) compared to those with low counts (green); notice the reduction in sites with frequency = 0 or 1 between cycle 12 and cycle 12 + heat shock.

We find 109,707 high quality SNPs in our sequenced populations that: (i) exceed 10X coverage per site; and (ii) are segregating among the four parental founder strains. The average coverage achieved in the haplotype founders was 52X, and the average coverage achieved in the recombinant populations was 82X. Supplementary Figure S1 illustrates how the alleles break down by founder; 86% of the alleles we identified are private to a single strain. Figure 2 shows the allele frequency distributions for these SNPs initially, after 6 cycles of outcrossing, after 12 cycles of outcrossing, and after 12 cycles of outcrossing including one round of heat shock at 55°C for 20 min. In the ancestral, “cycle 0” population, zero sites across the genome are fixed due to our filtering criteria. After 6 cycles of outcrossing, the number of fixed SNPs (with frequencies of 0 or 1) increases to 24,661 (22.4% of all SNPs), and after 12 cycles of outcrossing, the number of fixed SNPs increases to 48,490 (44.2% of all SNPs). In the population that received a heat shock treatment during the outcrossing protocol, we observed 37,859 fixed sites (34.5% of all SNPs). Thus, with increasing cycles of outcrossing, we observed dramatic increases in fixation across the genome, though the final heat shock step appears to decrease the number of fixed SNPs.

**FIGURE 3:**
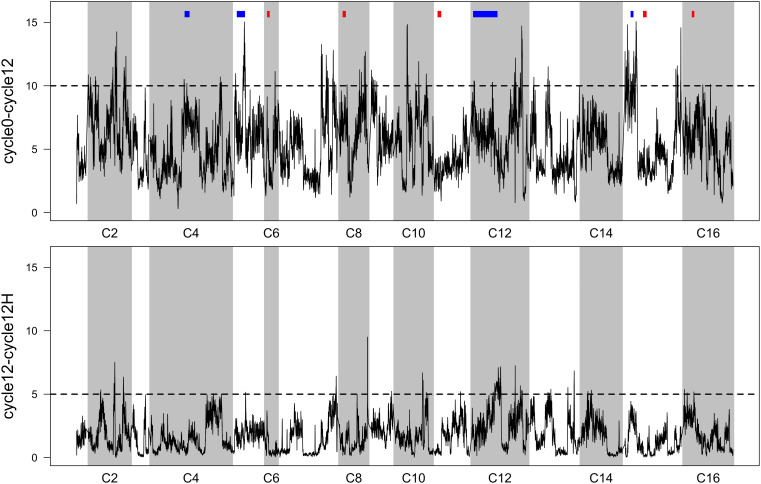
Sliding-window FET scores (*p*-values have been -log_10_ transformed). Top: per-SNP *p*-values comparing allele frequencies in the 4X population initially, vs. after 12 cycles of RSA and outcrossing. Previously reported QTL (9 regions from Cubillos et al., 2013) for outcrossing are shown in blocks above the peaks—blue indicates a significant overlap and red indicates no overlap. Bottom: per-SNP *p*-values comparing allele frequencies in the 4X population after 12 cycles of RSA and outcrossing, with and without a final heat-shock step. In both panels, regions that exceed the dotted line can be considered significantly differentiated (threshold derivations are described in Supplementary Figure S2).

Averaged *p*-values from FET tests were plotted as a function of genomic location (Figure 3). This reveals many localized “peaks” of significant *p*-values that might be associated with phenotypes relating to increased sporulation or mating efficiency (in the case of cycle 0 vs. cycle 12), or heat-shock (in the case of cycle 12 vs. cycle 12 H). To provide a threshold to interpret these data, we borrowed an approach from genome-wide association studies (GWAS) that involves plotting sorted observed *p*-values, and identifying a *y*-axis value that corresponds to a point where observed *p*-values clearly deviate from a uniform linear pattern. These sorted *p*-values and thresholds are provided in Supplementary Figure S2).

While we find it useful to provide a summary of regions that responded to 12 cycles of outcrossing, and to a single episode of heat-shock, we urge a conservative interpretation of these results. Given our lack of replication, we think that producing a list of regions/genes/GO terms from FET scores would not be informative. Instead, we favor comparing our results to a similar study (Cubillos et al., 2013), who identified candidate regions associated with sporulation, mating, and outcrossing in two experimental replicate populations. We cross-referenced our regions with those reported by Cubillos et al. (2013) (Supplementary Table S1A; these are the nine most significant candidates from their study). We do find some overlap; four regions on chromosomes 4, 5, 12, and 15 concur with these previously reported regions (Figure 3, top panel). Thus, these are fairly compelling candidates that might underlie traits that improve outcrossing.

**FIGURE 4:**
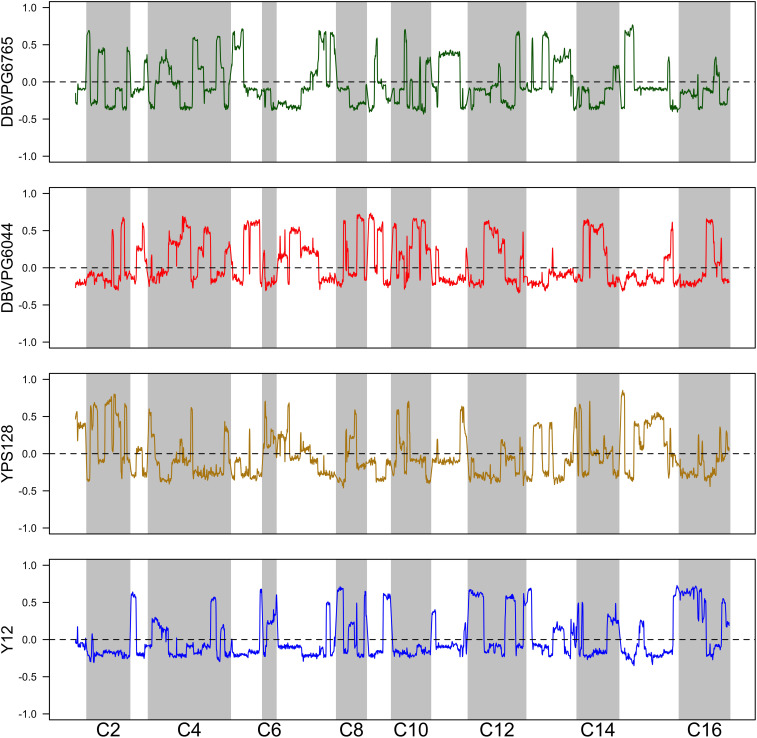
Haplotype differentiation observed in the 4X population after 12 rounds of outcrossing. The dotted line at zero provides a visual guide—frequencies that exceed this value increased over time, and those below this value decreased over time.

**FIGURE 5:**
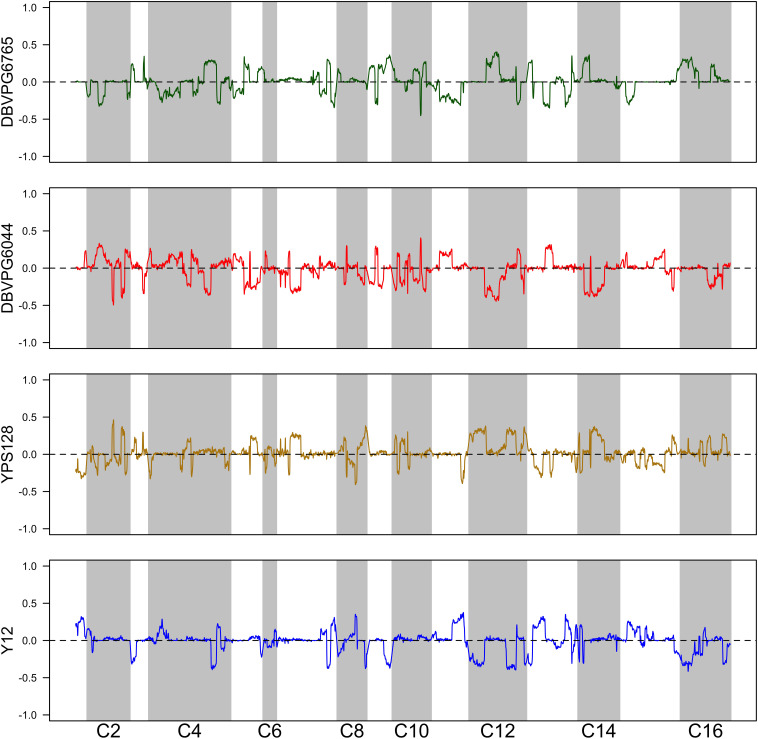
Haplotype differentiation observed in the 4X population after 12 rounds of outcrossing, with or without a 20 min heat-shock at 55°C. The dotted line at zero provides a visual guide—frequencies that exceed this value increased following heat-shock, and those below this value decreased following heat-shock.

Using the approach of Linder et al. (2020), we estimated haplotype frequencies in recombinant populations after 12 cycles of outcrossing, with and without a heat shock step. We generated 11,482 individual overlapping 30 kb windows for this haplotype estimation. Table 2 shows a summary of the mean genome-wide haplotype frequencies estimated in each population. We observed that the average haplotype frequencies of the YPS128 and DBVPG6765 founders increased by 2 and 0.7%, respectively, while they decreased in the DBVPG6044 and Y12 founders by 2 and 0.6%, respectively. This is notable in light of the fact that these two pairs of strains were crossed together first, during the construction of the 4-way population; therefore, these average increases and decreases likely reflect extensive linkage between these two pairs of genetic backgrounds. Haplotype frequencies for each founder are plotted as a function of genomic location in Supplementary Figures S3–S5. We found it especially helpful to visualize haplotype frequency changes associated with particular treatments by plotting the differences in frequencies between cycle 0 and 12 (Figure 4) and before and after heat-shock (Figure 5). Generally, we observed many haplotype regions changing in response to 12 rounds of outcrossing, and fewer regions responding to heat-shock.

**TABLE 2:** Haplotype frequencies estimated after 12 cycles of outcrossing with and without a heat shock step during the random spore enrichment protocol (55°C/20 min).

Haplotype	Frequency after 12 outcrossing cycles	Frequency after 12 outcrossing cycles + heat shock	Δ haplotype frequency
DBVPG6765	0.2372	0.2442	−0.0066
DBVPG6044	0.2915	0.2634	+0.0071
YPS128	0.2493	0.2709	+0.0216
Y12	0.2221	0.2155	−0.00656

**Numbers represent genome-wide averages.**

## Discussion

Our results suggest that heat shock may add value to RSA protocols in *S. cerevisiae*, because specific heat shock conditions kill vegetative cells but not spores. We observed the qualitative pattern that in all strains tested, cell viability in both vegetative and sporulated cultures decreases as the temperature of heat shock increases. The effect of heat shock duration was less obvious; while we generally expected that viability should also decrease with heat shock duration, this pattern was only observed at some temperatures, and only in some strains. In the isogenic strains, there was little quantitative variation in viability outside of the 50–55°C range; below this range viabilities generally approached 1, and above this range viabilities generally approached zero. By contrast, the 4-parent cross displayed considerably more quantitative variation in viability across heat shock temperatures and durations, especially in vegetative cultures. As the 4-parent cross is a highly recombinant population, the increased phenotypic variation observed is consistent with the idea that this population harbors a huge number of clonal lineages with specific genotypes that may underlie specific heat shock resistance phenotypes.

We were mainly interested in the viability differences between purely vegetative vs. sporulated cultures (which include mixes of vegetative and sporulated cells), as these differences may inform which heat shock conditions may lead to optimal enrichment of spores in mixed cultures. The general idea behind this rationale is that for any given heat shock condition, if vegetative culture viability is zero, and sporulated culture viability is greater than zero, we can reasonably conclude that sporulated cultures after heat shock do not include live vegetative cells. While this is a rather simplistic framework, we observed several specific heat shock conditions that resulted in near-zero viabilities in vegetative cultures and high viabilities in sporulated cultures. We observed at least one heat shock condition that resulted in this pattern in each strain tested, except for the Y12 strain, in which heat shock conditions that killed vegetative cultures also killed ∼90% of sporulated cultures. The YPS128 strain and the 4-parent cross exhibited viability patterns especially promising for spore enrichment by heat shock (*cf.* Figure 1). In these two strains we observed several heat shock conditions between 50 and 55°C with viability close to 100% in sporulated cultures and close to zero in vegetative cultures. We expected to observe variation in heat shock resistance phenotypes among strains, since the strains have such different genetic backgrounds and sites of origin. When we specifically looked for evidence of a “strain” effect, we found that it has a large effect for vegetative cultures and a more moderate (but still significant) effect on sporulated cultures. This could be evidence that response to heat shock is a more complex trait among vegetative cells than among sporulated cells; this interpretation is consistent with the idea that spores are more stress-resistant than vegetative cells, and perhaps this stress resistance is a feature of many strains. On the other hand, we acknowledge that our experiment was modest in size and perhaps not powerful enough to detect subtle differences between strains.

In addition to measuring viability following heat shock, we also assayed sporulation efficiency in these strains (Table 1). Others have previously characterized this strain-specific phenotype and some level of variation has been observed from study to study (e.g., Cubillos et al., 2009; Tomar et al., 2013). Given how sensitive sporulation efficiency is to preculture conditions, we were careful to replicate the exact conditions of our experiment. We find that strain YPS128 has high sporulation efficiency, 4X and Y12 have intermediate sporulation efficiency, and DBVPG6044 and DBVPG6765 have very low efficiencies. This is consistent with our viability estimates in some respects; for example, the strain ranking order observed in sporulation efficiencies (given in Table 1) was the same as that observed in the viabilities of sporulated cultures. In the highest sporulating strain (YPS128) it makes sense that spore viability is very high at temperatures where vegetative cell viability is low (e.g., 50–55°C), since very few vegetative cells should persist in sporulated cultures. Similarly, it makes sense that we observe low spore viability in our two strains that have very low sporulation efficiency, as there are likely few sporulated cells in these cultures. We find it very striking to compare genome-wide haplotype frequencies, especially in the initial 4-way cross (Supplementary Figure S3) with our observed sporulation efficiencies. Given the very low efficiencies observed in two of the four strains, one might expect to see low haplotype frequencies for these two strains, but instead all four are relatively evenly represented across the genome. This likely reflects our choice of initial strain pairs during the cross; F1 tetrads had a high-sporulating and a low-sporulating parent. We speculate that high levels of linkage, and perhaps also epistatic interactions, kept the haplotypes from low-sporulating strains segregating in the recombinant population, despite presumably having disadvantages for outcrossing.

As our major goal is to assess the potential for heat shock to improve RSA protocols, we generated a 4-parent yeast population using RSA with and without a heat shock step, and evaluated the genomic consequences of this step. Generally, and notably, we observed dramatic increases in fixation associated with our outcrossing/RSA protocol. Our RSA protocol included enzymatic, chemical, and mechanical agitation steps to kill vegetative cells and disrupt ascus walls. This perhaps renders it more severe than other RSA protocols, and leads to more unintended bottlenecking and fixation. By comparison, Cubillos et al. (2013) generated a 4-parent cross (called “SGRP4X”) that is identical in concept to our 4X population, though with different genotypes and drug resistance cassettes in the founding strains. The SGRP4X did not experience the same increases in fixation that we observe, which is likely due to multiple protocol differences. While more scrutiny is needed to determine exactly what led to this discrepancy, we speculate that a major source of variability between these two studies is the duration of sporulation. Our protocol involved a relatively short sporulation period (3 days) compared to the Cubillos study (10 days), and it is easy to imagine that the latter strategy led to higher overall sporulation efficiencies, and higher numbers of cells surviving the RSA protocol, as a result. The main result we wish to emphasize here is that despite widespread fixation after 12 cycles of outcrossing/RSA (43% of all SNPs fixed in the genome), a single episode of heat shock reduced this evident fixation (to 34% of all SNPs fixed in the genome). We interpret this reduction in fixed sites as evidence that heat shock may lead to increased mating between homozygous lineages. We speculate that our outcrossing/RSA protocol without heat shock leads to increases in clonal lineages that are alternately fixed for individual SNPs, perhaps due to homozygous vegetative cells “sneaking through” the protocol; if heat shock effectively kills most vegetative cells, this should lead to increased recombination and mating in the population. We recognize that such speculation should be viewed with caution, as we only observed this pattern in a single replicate population. On the other hand, we see few downsides to recommending heat shock as an effective and essentially no-cost addition to existing RSA protocols. As long as sporulation efficiency and spore viability are reasonably high in the strains being crossed (both phenotypes are straightforward to assess), our results suggest that heat shock events between 50 and 55°C, from 10 to 30 min, should increase the protocol’s effectiveness. Of course, our results also suggest that there is considerable variation among strains for phenotypes related to sporulation, mating, and heat stress, which have a genetic basis; thus, care should be taken to characterize these phenotypes in individual strains of interest prior to tailoring an RSA protocol to any cross between them. For investigators using high-throughput RSA protocols to generate and maintain highly recombinant yeast populations (e.g., for QTL mapping or experimental evolution work), heat shock appears a useful tool for preventing unwanted propagation of vegetative clonal lineages, with few viability consequences for recombinant spores.

## Data Availability Statement

All major data files (tables containing SNP and haplotype frequency data, viability results, etc.) will be made available through Dryad upon publication. The raw Illumina reads are also publicly accessible through NCBI SRA (PRJNA678990).

## Author Contributions

IK conceived of the project. IK and KM conducted the experiments. MB and IK analyzed the data and wrote the manuscript. All authors contributed to the article and approved the submitted version.

## Conflict of Interest

The authors declare that the research was conducted in the absence of any commercial or financial relationships that could be construed as a potential conflict of interest.

## Footnotes

1 www.R-project.org
