# Corrigendum: Heat Shock Improves Random Spore Analysis in Diverse Strains of *Saccharomyces cerevisiae*

**DOI:** 10.3389/fgene.2021.654893

**Published:** 2021-02-05

**Authors:** Molly K. Burke, Kaitlin M. McHugh, Ian C. Kutch

**Affiliations:** Department of Integrative Biology, Oregon State University, Corvallis, OR, United States

**Keywords:** random spore analysis, heat shock, sporulation, genomics, multiparent mapping population

In the original article, there was a mistake in Figure 2 as published. **The originally published figure had a panel out of order**. The corrected Figure 2 appears below.

**Figure 2:**
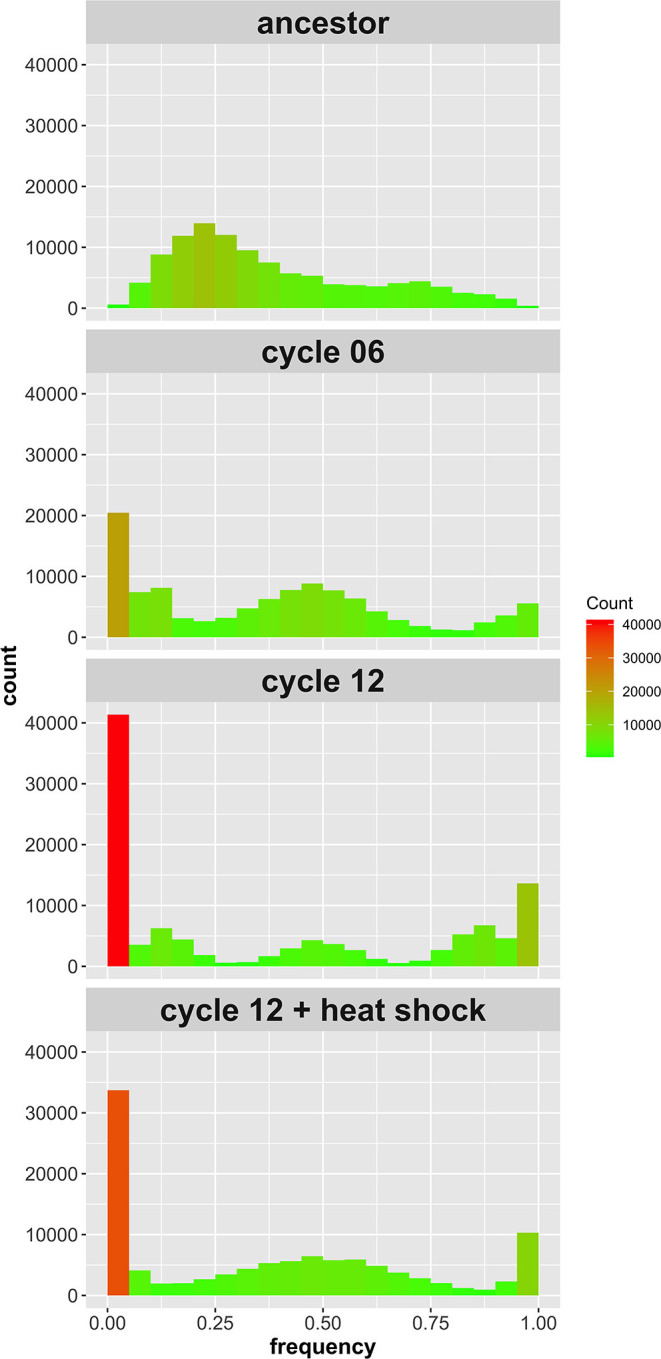
Site frequency spectrum of SNPs identified in the 4-parent population initially, after six outcrossing events, after 12 outcrossing events, and after 12 outcrossing events + a heat shock of 55°C for 20 min. Color in included in the histograms to emphasize the bins with high counts (red) compared to those with low counts (green); notice the reduction in sites with frequency = 0 or 1 between cycle 12 and cycle 12 + heat shock.

The authors apologize for this error and state that this does not change the scientific conclusions of the article in any way. The original article has been updated.

