# Supplementary material for: Heat Shock Improves Random Spore Analysis in Diverse Strains of Saccharomyces cerevisiae
Source: Front Genet. 2020 Dec 11;11:597482. doi: 10.3389/fgene.2020.597482 (PMC7759604; doi:10.3389/fgene.2020.597482)
Supplement: Supplementary file 1 [file Data_Sheet_1.PDF]

Supplementary Figures

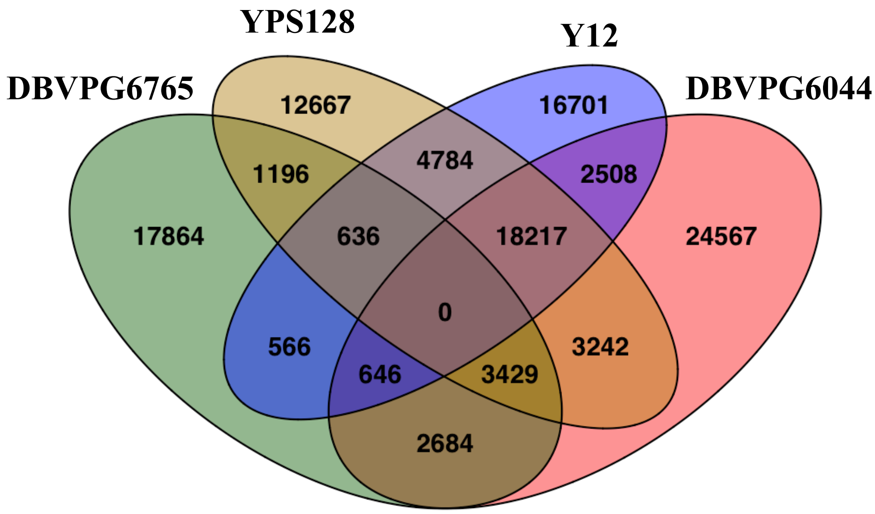

Figure S1. Venn diagram of SNPs observed in the 4X population (cycle 0), partitioned by founding strain. The numerical values above indicate specific alleles (alternative alleles to the reference S288C yeast genome) that are observed in each strain or combination of strains, out of a total of 109,707 alleles. ~86% of these alleles are private to a single founder.

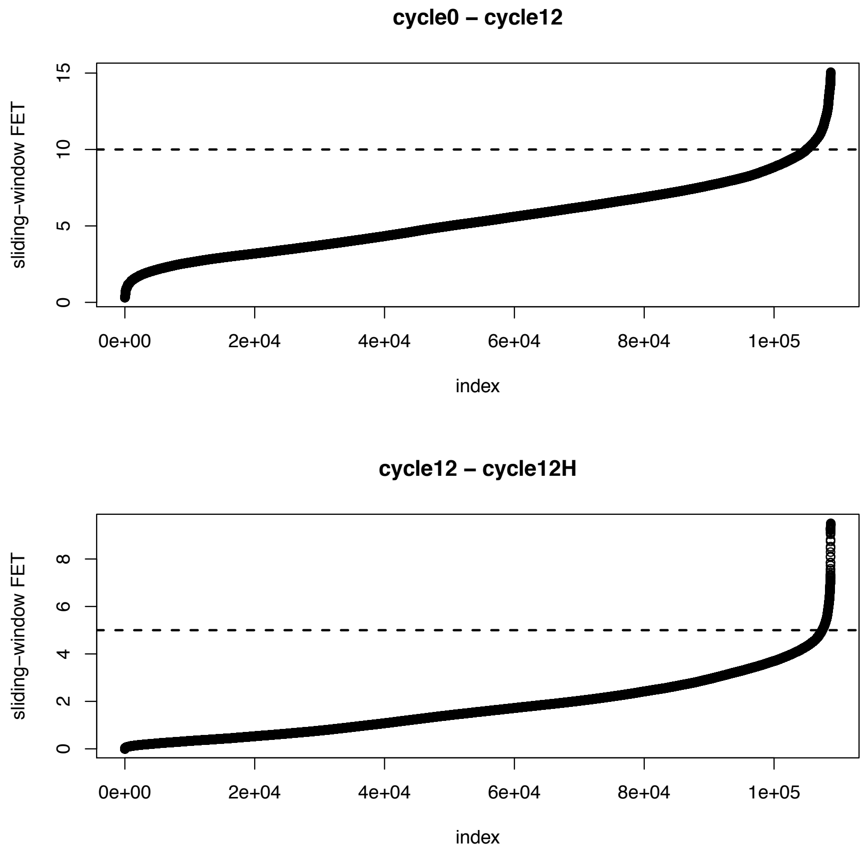

Figure S2. Empirical determinations of FET score significance thresholds. Observed p-values from FET scores (from all 109,707 SNPs) were sorted and plotted against the sorted null (uniformly distributed) *p*-values. The y-axis value where observed *p*-values clearly diverge from a linear pattern can be used as a rough significance threshold. These thresholds (y=10 for the cycle 0 – cycle 12 comparison, and y=5 for the cycle 12 – cycle12H comparison) are included in Figure 3 of the main text.

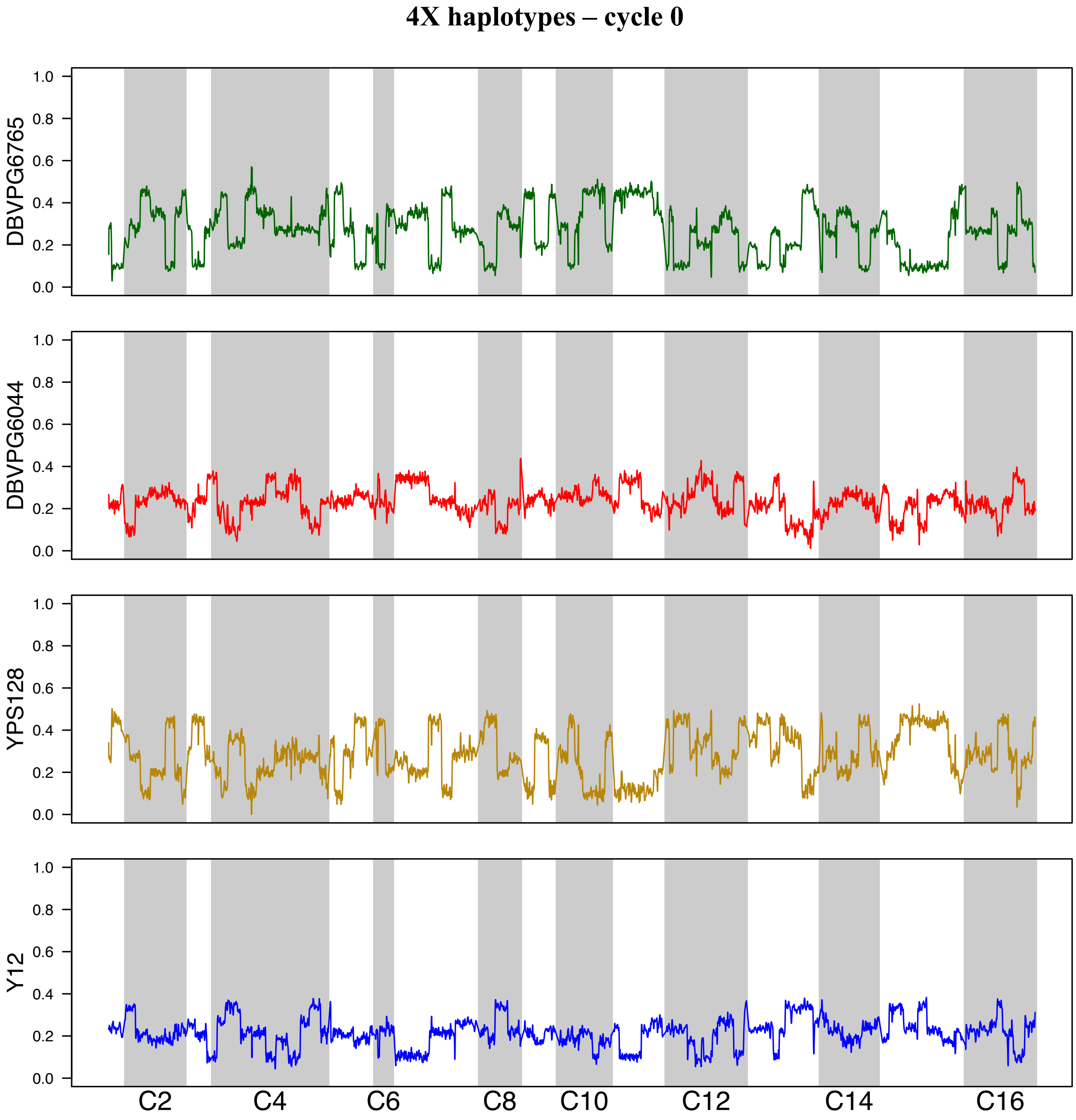

Figure S3. Genome-wide haplotype frequencies estimated in the 4X population initially (cycle 0). If all haplotypes were perfectly equally represented, each would occur with 0.25 frequency throughout the genome. Haplotypes were estimated with a 30kb window size and a step size of 1kb.

4X haplotypes – cycle 12

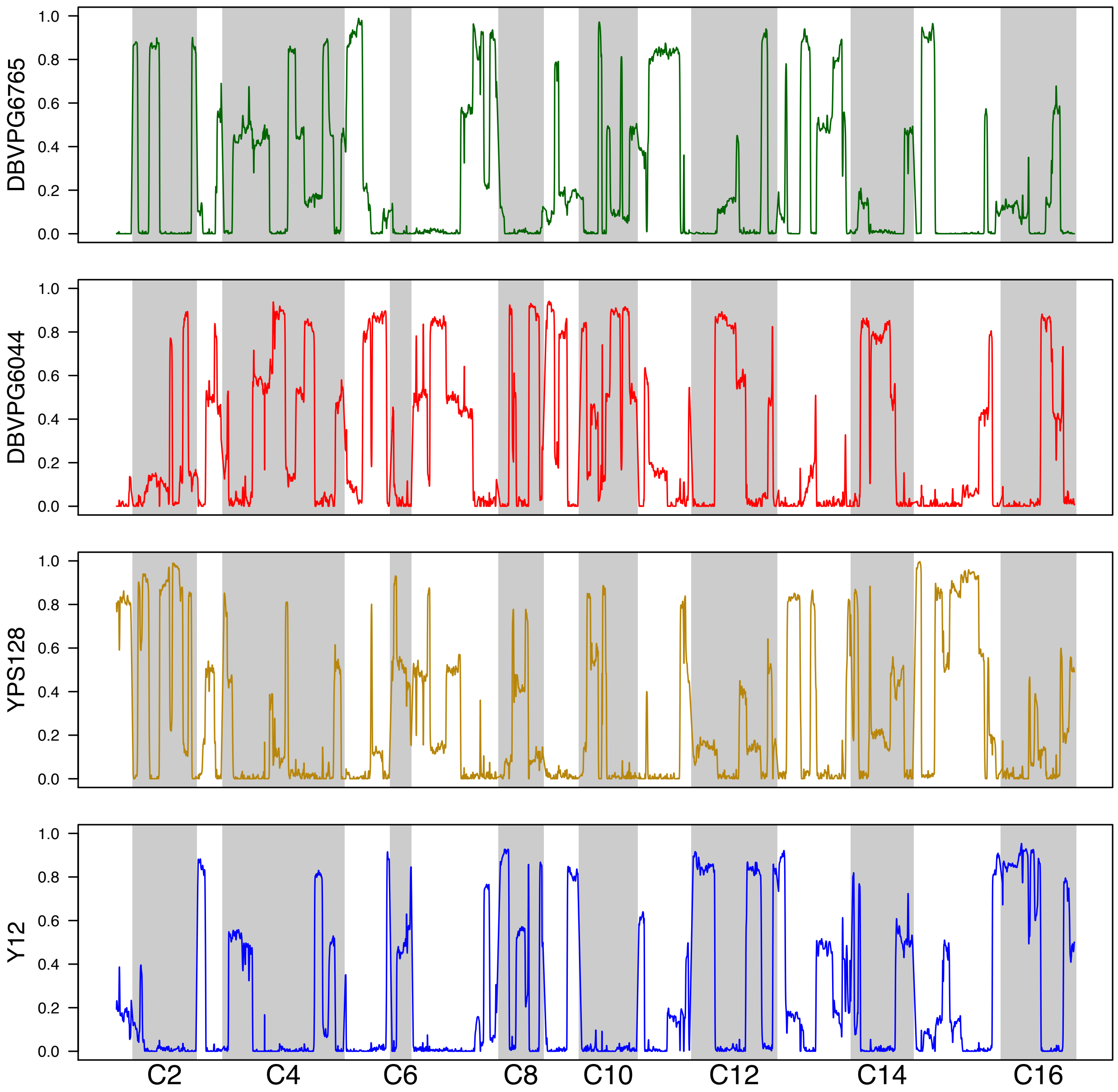

Figure S4. Genome-wide haplotype frequencies estimated in the 4X population after 12 RSA/outcrossing cycles. Haplotypes were estimated with a 30kb window size and a step size of 1kb.

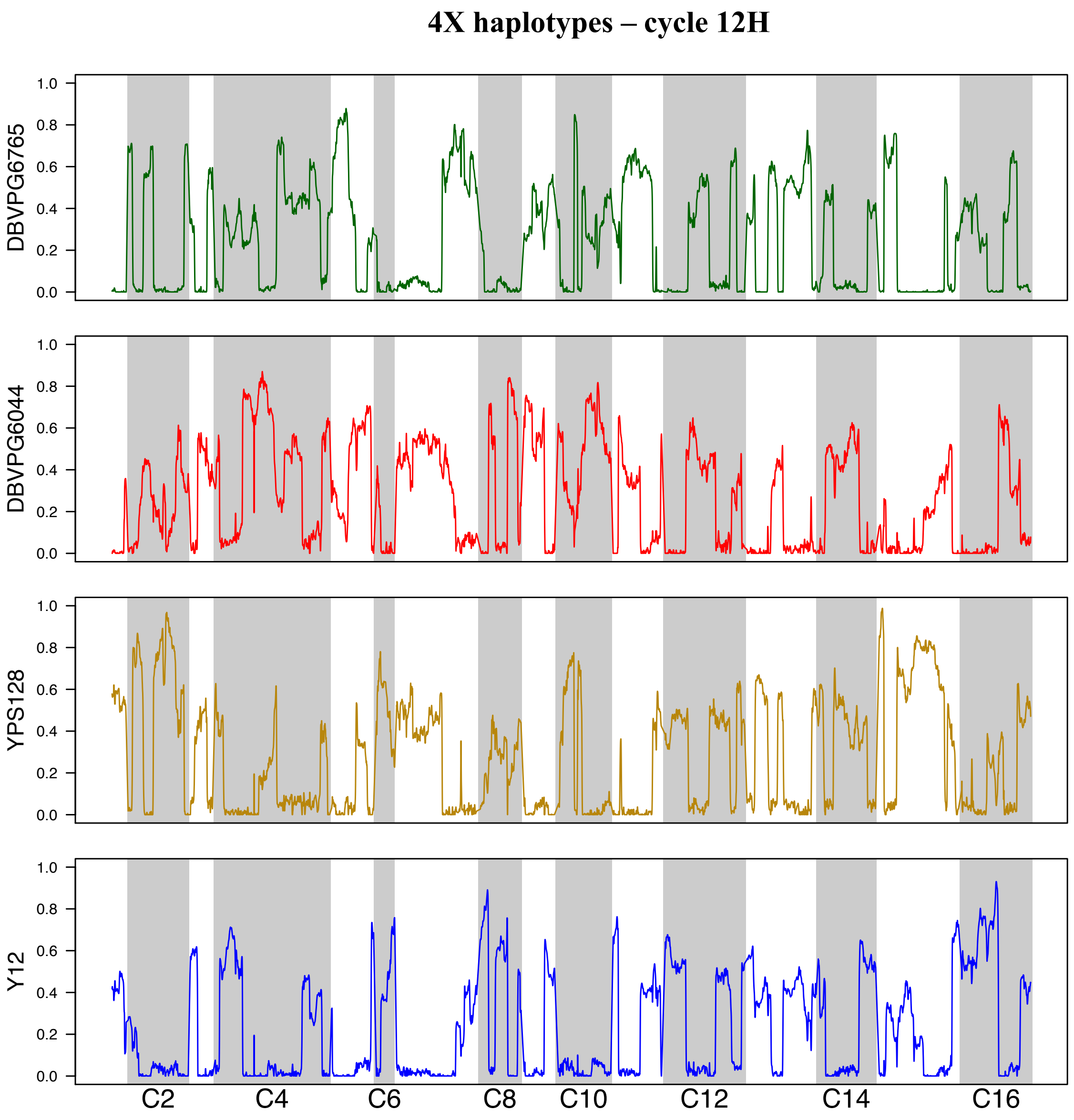

Figure S5. Genome-wide haplotype frequencies estimated in the 4X population after 11 RSA/outcrossing cycles, plus one cycle that included a heat-shock (55ºC for 20 mins). Haplotypes were estimated with a 30kb window size and a step size of 1kb.
